# Fractal analysis and assessment of lacunarity in mandibular osteoradionecrosis: a cross-sectional study with control group

**DOI:** 10.1590/1807-3107bor-2024.vol38.0114

**Published:** 2024-12-09

**Authors:** Natália Santos Barcelos, Cláudia Borges Brasileiro, Lucas Guimarães Abreu, Elismauro Francisco Mendonça, Sebastião Silvério Sousa-Neto, Sílvia Ferreira de Sousa, Ricardo Alves Mesquita, Patrícia Carlos Caldeira

**Affiliations:** (a) Universidade Federal de Minas Gerais – UFMG, School of Dentistry, Department of Oral Pathology and Surgery, Belo Horizonte, MG, Brazil.; (b) Universidade Federal de Minas Gerais – UFMG, School of Dentistry, Department of Pediatric Dentistry, Belo Horizonte, MG, Brazil.; (c) Universidade Federal de Goiás – UFGO, School of Dentistry, Department of Oral Pathology, Goiânia, GO, Brazil.

**Keywords:** Head and Neck Neoplasms, Fractals, Osteoradionecrosis, Radiotherapy, Radiography, Panoramic

## Abstract

The objective of this study was to evaluate the fractal dimension (FD) and lacunarity of the mandibular bone, comparing patients with and without osteoradionecrosis (ORN). In a cross-sectional study with a control group, 25 patients were included and divided into a case group (with ORN, n = 14) and a control group (without ORN, n = 11). A digital panoramic radiograph taken after the end of radiotherapy (RT) was evaluated for each patient. FD and lacunarity of the mandibular bone were determined using ImageJ software. Descriptive, bivariate, and ROC curve analyses were performed. Cohen's *d* effect sizes were calculated. Significance was established at p < 0.05. The mean FD and lacunarity values were not significantly different between the groups. The area under the curve for FD and lacunarity were 0.579 and 0.661, respectively. The cut-off point for FD was ≤1.1714 and for lacunarity, > 0.3821, correctly classifying the majority of cases and controls. Most participants in the case group (63.6%) had a FD ≤ 1.1714 and the majority of participants in the control group (63.6%) had a FD >1.1714 (p = 0.395). For lacunarity, most individuals in the case group (72.7%) had a value > 0.3821 and most participants in the control group (63.6%) had a value ≤ 0.3821 (p = 0.198). In conclusion, the FD and lacunarity values did not show statistically significant differences between patients with and without ORN. However, the moderate and large magnitude of the effects seem to indicate that the results may be clinically relevant.

## Introduction

Head and neck cancer (HNC) affects the upper aerodigestive tract and is considered a public health problem in many countries.^1,2^ Surgery, radiotherapy (RT), chemotherapy, and a combination of these treatments are therapeutic modalities frequently used for HNC. The effect of radiation therapy occurs when the ionizing radiation damages the deoxyribonucleic acid (DNA), inducing cell death or loss of the proliferative capacity in cells with a high turnover rate.^3,4^

RT may lead to acute or chronic oral complications.^5^ One of the most severe and difficult to manage complications is osteoradionecrosis (ORN), a late complication of RT. It is clinically characterized as an exposure of devitalized bone that does not heal for three or more months in the absence of the neoplasm.^6,7^ The etiopathogenesis of ORN remains controversial, but the most accepted theory is the association with changes in metabolism and cellular activity of irradiated bone, as described by Marx.^8^ Radiographically, a low density of the local bone structure, osteolytic areas, and cortical disruption are observed.^9^

Panoramic radiographs are frequently used in dentistry because of their low cost, they reveal bone changes, allow analysis of trabecular bone, and require low exposure to radiation.^10^ One of the methods currently available for bone evaluation using panoramic radiographs is fractal analysis, a mathematical method that describes and analyses complex forms and structural patterns based on fractals. The numerical expression of fractal analysis is the fractal dimension (FD), which can be calculated by the box-counting algorithm.^11–14^ Lacunarity is another fractal feature that characterizes the texture of structures, obtained by means of the measurement of the spatial distribution and gap arrangement in the image.^15–17^

Fractal analysis of digital images has been successfully used to evaluate pathological and physiological changes in bone architecture, such as changes observed in osteoporosis, osteogenesis imperfecta, type 2 diabetes mellitus, periodontal disease, and osteonecrosis.^18^ FD may be increased or reduced depending on the bone architectural complexity.^17,19 20^ However, there is a lack of studies using this method for the evaluation of some lesions, including ORN of the jaws. Considering the controversy in the literature regarding the best period for surgical intervention in patients irradiated in the head and neck area, the lack of predictive factors for ORN, the morbidity, and the unpredictable response to treatment, all patients treated with RT are considered at high risk for ORN.^21^ For this reason, studies that identify predictive factors for ORN, especially with non-invasive methods, are highly encouraged.

The aim of this study was to evaluate the FD and lacunarity of the mandibular bone in individuals who received radiation therapy for head and neck cancer, by comparing patients who developed ORN with those who did not.

## Methods

### Study population

The protocol of this cross-sectional study with a control group was in agreement with the Declaration of Helsinki and approved by the Research Ethics Committee of the *Universidade Federal de Minas Gerais* (certificate number: 30560820.9.0000.5149). This study was reported following the Strengthening the Reporting of Observational studies in Epidemiology’ (STROBE) guidelines.^22^

The sample was selected at *Universidade Federal de Minas Gerais* and *Hospital de Câncer Araújo Jorge da Associação do Combate ao Câncer em Goiás*, Brazil. Inclusion criteria were patients who had received three dimensional (3D) conformal RT for head and neck cancer treatment, with a panoramic radiograph taken after the end of treatment. The following exclusion criteria were applied: radiation dose lower than 60 Gy, age < 40 and > 70 years, reirradiation, use of bisphosphonates or other bone-modifying drugs (antiresorptives and antiangiogenic agents), and poor image quality of panoramic radiograph.

Data on demographics, oncological treatment, and oral health were collected from the electronic medical records database. The selected patients were classified into two groups according to the occurrence of osteoradionecrosis: case group (n = 14), composed of patients who had developed ORN, and control group (n = 11), consisting of individuals who had not developed ORN. Two dentists who are specialists in dental care for oncological patients (P.C.C. and E.F.M.) determined the diagnosis of ORN. The clinical criterion used was an exposure of devitalized bone that does not heal for three or more months in the absence of a neoplasm^6,7,9^. The radiographic aspects observed were low bone density, osteolytic areas, and/or cortical disruption^9^. The groups were paired by age and sex. The sample size was calculated (specified power = 90%) based on the results reported by Sahin et al*.* ^13^

### Fractal analysis

The fractal analysis of the digital panoramic radiographs was performed by two trained observers (C.B.B and N.S.B) using the FracLac plugin of ImageJ software (National Institutes of Health, Bethesda, USA). As described by White and Rudolph,^23^ all images were converted to 8 bits, enabling each pixel to display a maximum of 256 shades of gray. The regions of interest (ROIs) were chosen according to the groups.

**Figure 1:**
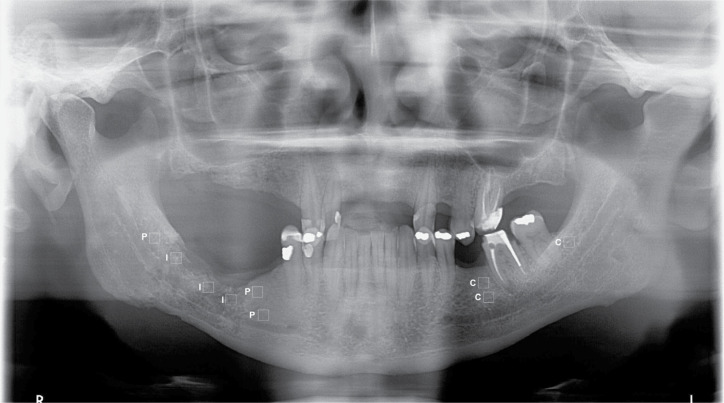
Location of intralesional (I), perilesional (P), and contralateral (C) regions of interest (ROIs).

For the case group, three ROIs of 50 x 50 pixels were delimited in mandibular bone: intralesional, perilesional, and contralateral side of the ORN (Figure 1). The unaffected side was set as the contralateral side. In this region, ROIs were delimited in alveolar bone, above the mandibular canal. Cortical bone, lamina dura, root structures, oblique line of mandible, and mandible angle were not included in ROIs (Figure 1).

In the control group, three ROIs with the same size were selected in the mandible side corresponding to the irradiated tumor. In the absence of this information, bilateral measurement was performed. ROIs were delimited in alveolar bone, upper to mandibular canal. Cortical bone, lamina dura, root structures, oblique line of mandible, and mandible angle were not included in ROIs.

In both groups, the mean was calculated to obtain the final value and all measurements were performed in the anatomical region from the lower left first premolar to the lower left third molar and from the lower right first premolar to the lower right third molar. The anatomical region from the left canine to the lower right canine was not included in ROIs to avoid overlapping the spine.

**Figure 2:**
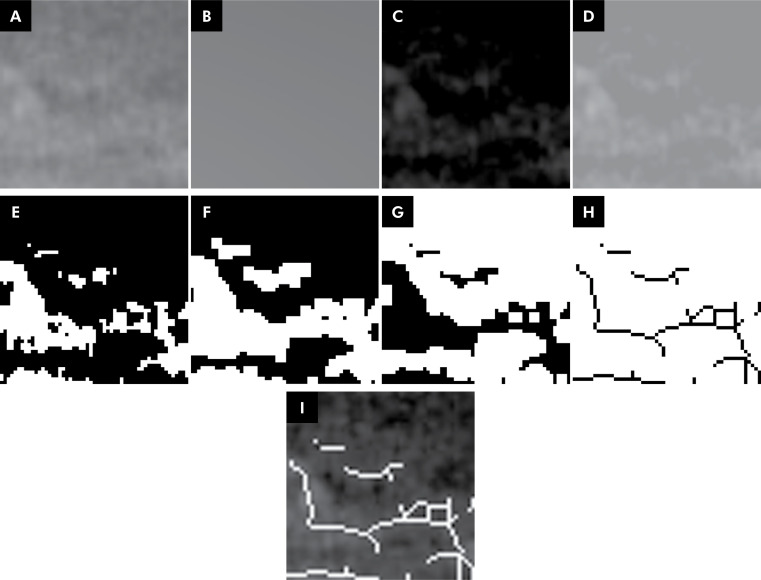
Image processing in fractal analysis.

Imaging processing was then performed as described by White and Rudolph^23^ and Palma et al.^10^ (Figure 2). First, the selected ROI was duplicated and blurred by a Gaussian filter (sigma = 35 pixels) to retain only large density variations of the structures. The obtained image was subtracted from the original images. To differentiate trabecular bone from medullary spaces, 128 levels of gray were added. The image was then made binary. The image was dilated and eroded to reduce noise and keep the contours evident. Next, the image was inverted (marrow spaces as white areas and trabecular bone as black areas), skeletonized, and the ROI of the skeletonized image was overlaid with the initial image to verify the match. Finally, the box-counting method was applied to determine the fractal dimension and lacunarity in the skeletonized image (Figure 2H).

A) Duplication of the region of interest to be analyzed; B) Application of the Gaussian blurred filter; C) Subtraction of image B from image A; D) Addition of 128 gray values to each pixel; E) Binarization; F) Dilation and erosion; G) Inversion; H) Skeletonization; I) Result of the superposition of H over A.

### Statistical analysis

The collected data were organized and coded in a database and statistical analyses were performed using the Statistical Package for the Social Sciences^®^, version 19.0 (IBM Inc., Armonk, USA). Clinical and demographic data were analyzed descriptively. The Shapiro-Wilk test demonstrated that quantitative data had a normal distribution. Therefore, comparisons of FD and lacunarity between groups were performed using Student's *t*-test. Mean and standard deviation (SD) were determined. For all tests, p-values < 0.05 were considered statistically significant. Cohen's *d* effect sizes (ES) were also calculated dividing the mean difference between groups by the pooled standard deviation. Cohen's *d* around 0.20 denoted a small effect, 0.5 indicated a moderate effect, and 0.80 denoted a large effect.^24^ To determine a cut-off value for FD and lacunarity to distinguish individuals who had or had not developed ORN, receiver operator characteristics (ROC) curves^25^ were calculated with the MedCalc software (MedCalc Software BVBA, Ostend, Flanders, Belgium). In the dataset, FD and lacunarity were entered as continuous variables. Occurrence of ORN was entered as a dichotomous variable (case group = with ORN and control group = without ORN). The area under curve (AUC), sensitivity (true positive), specificity (true negative), and 95% confidence intervals (CI) were determined. The Youden index was calculated to define the cut-off point for FD and lacunarity to maximize sensitivity and specificity, that is, (sensitivity + 1 - specificity).^26,27^

Using the cut-off points and the two continuous scales (FD and lacunarity) converted to dichotomous tests, comparisons between cases and controls were performed with chi-square tests. Statistical significance was set at p < 0.05. Odds ratio was also calculated as an effect size measure. Odds ratio close to 1.50 (small effect), 2.50 (moderate effect), and 4.00 (large effect) were equivalent to Cohen's *d* around 0.20 (small), 0.5 (moderate), and 0.80 (large), respectively.^24,28,29^

## Results

Among the 25 individuals selected for the study, 22 were males (88.0%) and three were females (12.0%). Twenty-three (92.0%) patients had undergone chemotherapy associated with RT. Clinical and demographic features of participants are shown in Table 1 and Table 2.

**Table 1:** Clinical features of participants included in the case (n = 14) and control (n = 11) groups.

Variable		Control	Case
		n (%)	n (%)
Histological type			
	Squamous cell carcinoma	11 (100.0)	12 (85.7)
	Carcinoma not specified	00 (00.0)	02 (14.3)
Tumor location			
	Oropharynx	07 (63.6)	08 (57.1)
	Oral cavity	04 (36.4)	05 (35.7)
	Salivary glands	00 (00.0)	01 (07.1)
Smoking habit			
	Yes, current	05 (45.5)	05 (35.7)
	Yes, previous	05 (45.5)	06 (42.9)
	No	01 (09.1)	02 (14.3)
	Missing information	00 (00.0)	01 (07.1)
Alcohol use			
	Yes, current	02 (18.2)	06 (42.9)
	Yes, previous	06 (54.5)	05 (35.7)
	No	03 (27.3)	02 (14.3)
	Missing information	00 (00.0)	01 (07.1)
Tooth extraction			
	Mandible	04 (36.4)	06 (42.9)
	Maxilla	01 (09.1)	00 (00.0)
	Mandible and maxilla	01 (09.1)	03 (21.4)
	Not performed	05 (45.5)	05 (35.7)
Osteoradionecrosis			
	Mandible	Not applicable	12 (85.7)
	Maxilla and mandible	Not applicable	02 (14.3)

**Table 2:** Clinical and demographic features of participants included in the case (n = 14) and control (n = 11) groups.

Variable	Control		Case	
	Mean (SD)	Minimum - Maximum	Mean (SD)	Minimum - Maximum
Age	59.82 (1.726)	48–67	58.79 (1.684)	48 – 67
Radiotherapy sessions	34.36 (0.907)	30–42	33.43 (0.732)	30–40
Total dose (Gy)	67.27 (1.054)	60–70	66.50 (0.999)	60–70
Time between radiotherapy completion and radiography acquisition (months)	22.18 (8.885)	00–95	50.79 (9.666)	07–121
*Time between radiotherapy completion and tooth extraction (months)	11.33 (4.91)	03 – 20	45.07 (13.01)	0.63–118
Time between radiotherapy completion and osteoradionecrosis (months)	Not applicable	Not applicable	42.50 (8.426)	06 – 121

* Data available for 3 patients of the control group and 8 patients of the case group.

**Figure 3:**
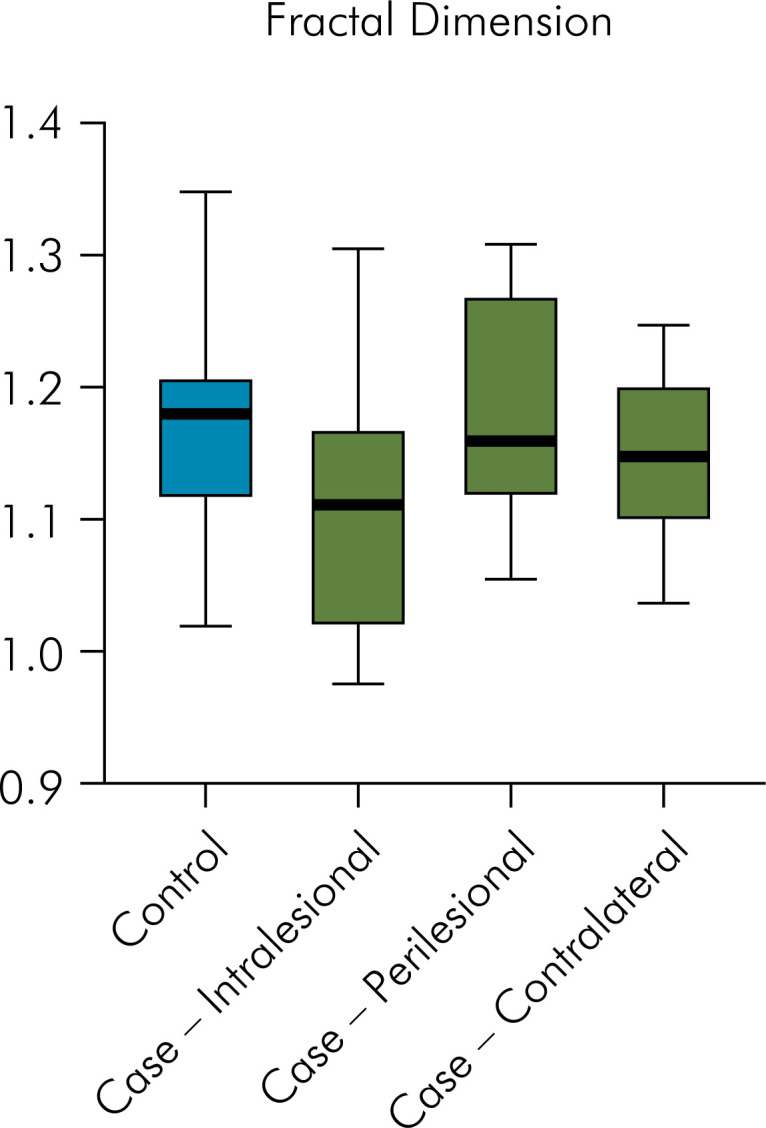
Graphical representation of fractal dimension (FD) values according to regions of interest (ROIs) with no statistically significant differences.

For the FD, no significant differences between the control group (mean = 1.167, SD = ± 0.090) and the intralesional values (mean = 1.106, SD = ± 0.095, p = 0.118, ES = 0.66), the perilesional values (mean = 1.181, SD = ± 0.083, p = 0.708, ES = 0.16), and the contralateral values (1.148, SD = ± 0.061, p = 0.554, ES = 0.25) of the case group were observed (Figure 3).

**Figure 4:**
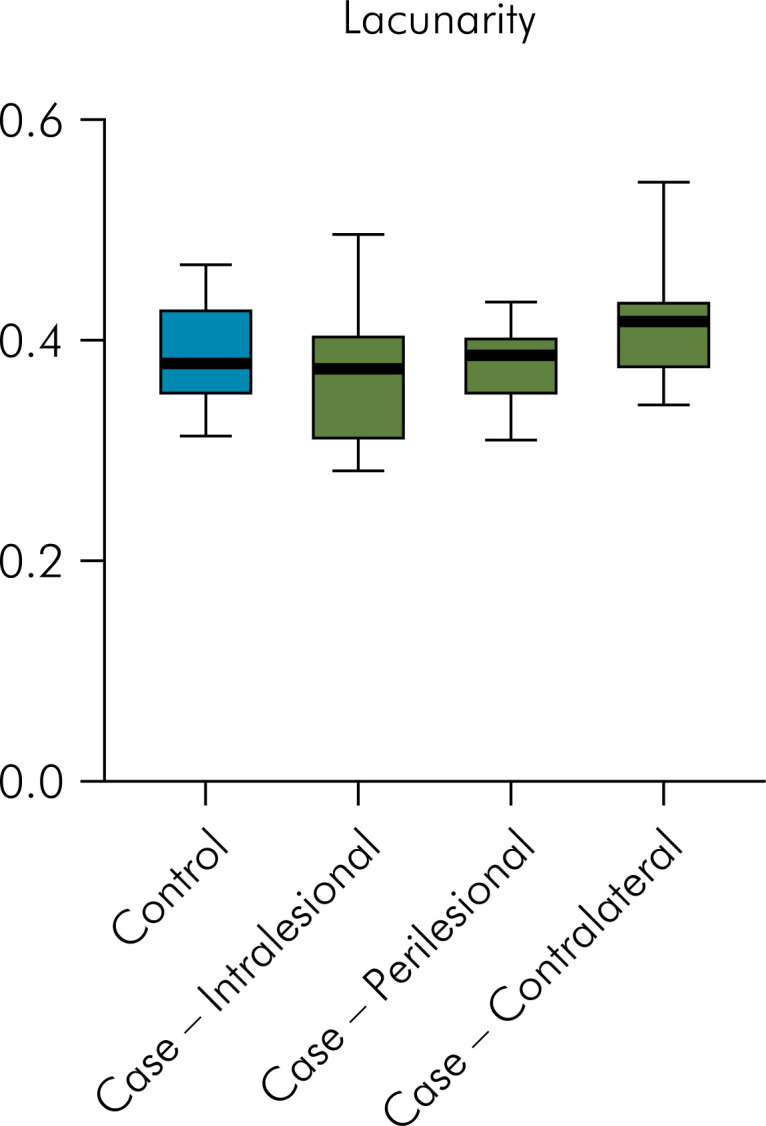
Graphical representation of the lacunarity values according to regions of interest (ROIs) with no statistically significant differences.

For the lacunarity values, no significant differences between the control group (mean = 0.385 SD = ± 0.048) and the intralesional region (mean = 0.366 SD = ± 0.061, p = 0.418, ES = 0.35), the perilesional region (mean = 0.375 SD = ± 0.036, p = 0.585, ES = 0.23), and the contralateral region (mean = 0.418 SD = ± 0.059, p = 0.164, ES = 0.62) of the case group were observed (Figure 4).

**Figure 5:**
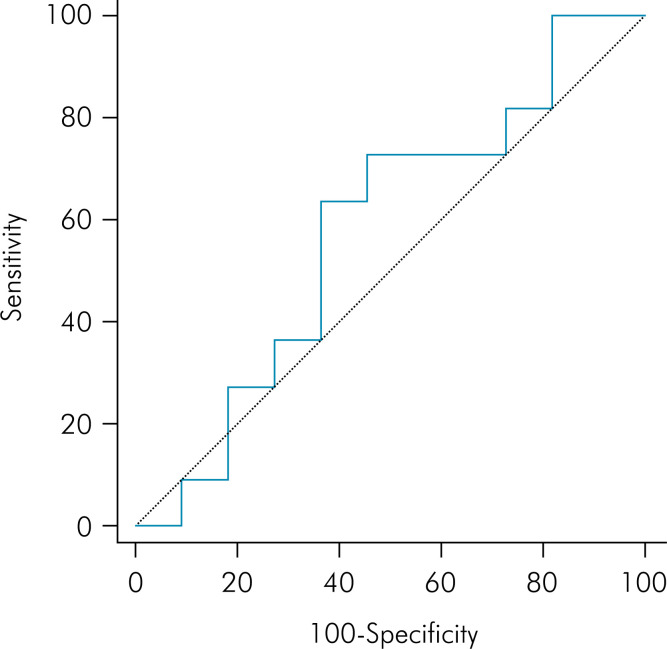
Receiver operating characteristic curve of fractal dimension (FD).

**Figure 6:**
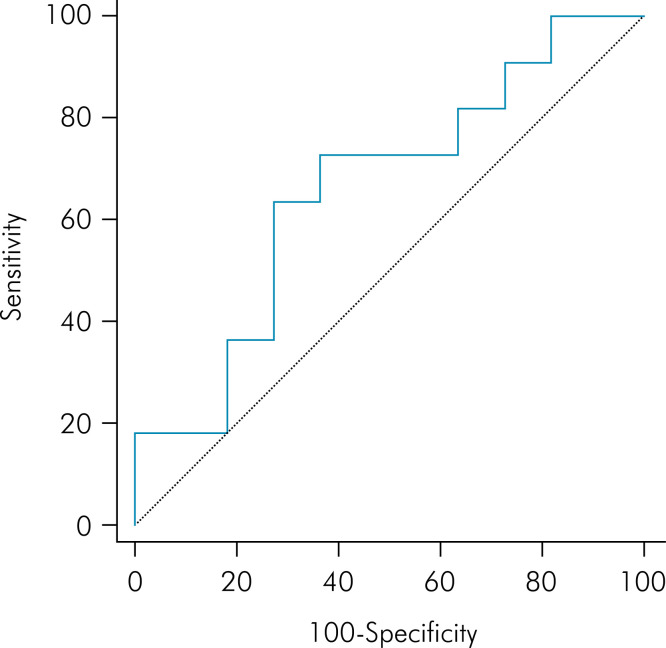
Receiver operating characteristic curve of lacunarity.

The AUC for FD was 0.579 (Figure 5) and for lacunarity was 0.661 (Figure 6). The cut-off point found for FD was ≤1.1714 and for lacunarity, > 0.3821 (Table 3). Using these values for the analysis, the majority of participants in the case group (63.6%) had a FD ≤1.1714 and the majority of participants in the control group (63.6%) had a FD >1.1714. Even though no significant difference was observed (p = 0.395), the odds ratio was 3.06, indicating a moderate ES. In the same way, most individuals in the case group (72.7%) had a value of lacunarity >0.3821 and most participants in the control group (63.6%) had a lacunarity value ≤0.3821. Although no significant difference was observed (p = 0.198), the odds ratio was 4.66, indicating a large ES (Table 4).

The area under the curve of fractal dimension is 0.579 (95% confidence interval: 0.352–0.783). The Youden index of this analysis is 0.272 with a specificity of 63.6% and a sensitivity of 63.6%. The cut-off point is ≤ 1.1714.

The area under the curve of lacunarity is 0.661 (95% confidence interval: 0.431–0.846). The Youden index of this analysis is 0.363 with a specificity of 63.6% and a sensitivity of 72.7%. The cut-off point is > 0.3821.

**Table 3:** Evaluation of the area under the curve, confidence interval (95%), sensitivity, specificity, Youden index, and cut-off point for fractal dimension and lacunarity.

Variable	Area under the curve	Confidence interval (95%)	Sensitivity (%)	Confidence interval (95%)	Specificity (%)	Confidence interval (95%)	Youden index	Cut-off point
Fractal dimension	0.579	0.352–0.783	63.6	30.8– 89.1	63.6	30.8– 89.1	0.272	≤ 1.1714
Lacunarity	0.661	0.431–0.846	72.7	39.0– 94.0	63.6	30.8– 89.1	0.363	>0.3821

**Table 4:** Distribution of cases and controls according to the cut-off point for fractal dimension and lacunarity.

Variable		Control	Case	Odds ratio	p-value *
		n (%)	n (%)		
Cut-off point for fractal dimension					
	> 1.1714	07 (63.6)^A^	04 (36.4)^B^	3.06	0.395
	≤ 1.1714	04 (36.4)^C^	07 (63.6)^D^		
Cut-off point for lacunarity					
	≤ 0.3821	07(63.6)^A^	03 (27.3)^B^	4.66	0.198
	> 0.3821	04 (36.4)^C^	08 (72.7)^D^		

* Pearson's test.

A = true negatives;

B = false negatives;

C = false positives;

D = true positives.

## Discussion

In the present study, fractal analysis of the mandible bone was performed in individuals who underwent RT for head and neck cancer, comparing patients with and without ORN, looking for a method that could assist in predicting ORN occurrence. To our knowledge, no previous research has used this noninvasive method to investigate ORN, a late and severe complication of RT. The establishment of significant cut-off points for FD and lacunarity to differentiate irradiated patients who had developed ORN from those without ORN was not possible. However, most participants in both groups were correctly classified. Despite the lack of statistical significance, the moderate and large effect sizes obtained demonstrate that the findings of this study may be meaningful and clinically relevant,^30^ allowing us to hypothesize that fractal analysis may help in identifying patients with a higher risk of developing ORN. Future studies with larger samples should test and validate this hypothesis, considering the oncological and clinical characteristics of the patients, particularly the already established risk factors for ORN^21^. In this sense, a nomogram could be further delineated, taking into account the clinical features along with radiomics data.^31^

In ORN areas, the trabecular bone was heterogeneous with different bone density between the affected region (radiolucent and radiopaque areas) and the unaffected region (uniform bone). However, the mean values of FD and lacunarity in the case and control groups were similar and had no statistically significant differences. According to Demirbas et al.,^11^ different sets of fractals may have the same FD values while exhibiting different textures, although more complex textures are specific to structures with higher FD values, which can explain this result. Findings regarding FD values in lesions with changes in bone density, such as osteoporosis or decalcifications, are conflicting in the literature.^11,14^ Some authors found a positive correlation between fractal analysis and bone mineral density, while others found inversely proportional values, as reported by Kato et al.,^14^ attributing the discrepancies to the methods used in the studies.

The literature on radiomics of irradiated mandibular bone is quite incipient thus far. Palma et al.^10^ showed a slight, albeit statistically significant (p = 0.0495) reduction of FD of the mandibular bone after RT (1.3 ± 0.1) compared to FD before RT (1.4 ± 0.1). The study employed a ROI of 100 x 100 pixels located unilaterally in the right angle of the mandible, below the mandibular canal, and posterior to the molar region.

Another study^13^ reported that, overall, patients with medication-related ORN of the jaws (MRONJ) at an early stage had lower mean FD of mandibular bone than those with MRONJ at an advanced stage. That study found no statistically significant difference between groups, except for the superior region of the mandibular canal on the distal side of mental foramen. Although the etiopathogenesis of MRONJ differs from that of ORN,^32,33^ the radiographic findings of both lesions can be similar and overlap in some stages of lesion development, with osteolysis and radiolucent areas of bone destruction.^34,35^ Further research should investigate the radiomics of different stages of ORN.

The pathogenesis of ORN is still not fully understood, but tooth extractions are considered a major risk factor.^1,36,37^ The mandibular bone is usually more affected than the maxillary bone, as found herein, probably due to the lower vascularity and blood supply of mandible.^7,38^ In the present sample, most patients from the ORN group had tooth extractions, but a significant percentage (35.7%) had none. Also in the present study, 54.6% of patients without ORN have had tooth extractions. The low frequency of post-extraction ORN in our study is similar to that in Saito et al*.* ^39^ Among 32 patients evaluated, only nine developed ORN after tooth extractions. Factors other than tooth extractions also influence the occurrence of ORN, such as tumor site, radiation modality and dose, surgical extraction technique, and the time between RT ending and tooth extraction.

In the present study, the average time interval between RT completion and tooth extraction was 3.7 years for the ORN group and 0.9 year for the control group. This seems to corroborate the findings of previous studies^9,39,40^ that reported a higher frequency of trauma-related mandibular ORN during the time interval corresponding to the second peak incidence of the bimodal pattern of ORN development described by Marx et al^41^. The second peak occurs between 2 and 5 years after the completion of RT. Similarly, the first peak occurs in the first 3 months after the end of RT, and in the control group, tooth extraction was performed between 3 months and 1.6 years, remaining outside the susceptible periods.

Several factors can affect bone metabolism and consequently lead to trabecular irregularity, influencing the values of DF and lacunarity.^19^ Some of these variables are age, tumor location, total radiation dose, and the time between the end of radiotherapy and radiographic acquisition. With advancing age, both cortical and medullary bone undergo remodeling, with bone resorption being greater than bone formation.^19^ The location of the tumor influences the exposure and total radiation dose received by the bone and soft tissues adjacent to the lesion. As mentioned in a previous study,^10^ the FD of the mandibular bone showed a discrete reduction in DF after RT compared to pre-RT values. The literature also reports the development of jaw bone sclerosis following radiotherapy,^42,43^ probably resulting from an increased number of bone trabeculae. Chan et al.^43^ pointed out that after the initial radiation-induced depletion of osteoblasts and consequent bone resorption, there is bone deposition by residual osteoblasts as an attempt to repair bone. Additionally, tobacco and alcohol act as irritants to the mucosa and inhibit healing, increasing the risk of ORN development.^3,44,45^ In the present study, age was matched between cases and controls, and tumor location and total radiation dose were used as sample selection criteria to minimize interference in the results.

Acquiring images with different types of equipment can be a limitation in fractal analysis, minimized by prior image processing. Nonetheless, most studies reveal that FD is not affected by variations in X-ray exposure and small variations in beam alignment. Other limitations of fractal analysis are the lack of standardization of the size and location of the ROI. The ROI size may vary according to the size of the studied structure and the location of the ROI is influenced by the type of exam, especially to avoid overlapping structures on the image.^10,17,46^

## Conclusion

In conclusion, the FD and lacunarity values were not significantly different between patients with and without ORN. However, the moderate and large magnitude of the effects seem to indicate that the results may be clinically relevant. The applicability of the cut-off values to assess the risk for ORN development should be further explored in future research, especially with larger sample sizes.

## References

[1] Kawashita Y, Soutome S, Umeda M, Saito T (2020). Oral management strategies for radiotherapy of head and neck cancer. Jpn Dent Sci Rev.

[2] Louredo BVR, Lima-Souza RA, Pérez-de-Oliveira ME, Warnakulasuriya S, Kerr AR, Kowalski LP (2024). Reported physical examination methodologies for screening of oral cancer and oral potentially malignant disorders: a systematic review. Oral Surg Oral Med Oral Pathol Oral Radiol.

[3] Rivero JA, Shamji O, Kolokythas A (2017). Osteoradionecrosis: a review of pathophysiology, prevention and pharmacologic management using pentoxifylline, α-tocopherol, and clodronate. Oral Surg Oral Med Oral Pathol Oral Radiol.

[4] Moore C, McLister C, Cardwell C, O'Neill C, Donnelly M, McKenna G (2020). Dental caries following radiotherapy for head and neck cancer: A systematic review. Oral Oncol.

[5] Faustino IS, Georgaki M, Santos-Silva AR, Vargas PA, Lopes MA (2022). Head and neck radiotherapy leading to extensive late oral soft-tissue necrosis. Oral Oncol.

[6] Harris M (1992). The conservative management of osteoradionecrosis of the mandible with ultrasound therapy. Br J Oral Maxillofac Surg.

[7] Moon DH, Moon SH, Wang K, Weissler MC, Hackman TG, Zanation AM (2017). Incidence of, and risk factors for, mandibular osteoradionecrosis in patients with oral cavity and oropharynx cancers. Oral Oncol.

[8] Marx RE (1983). Osteoradionecrosis: a new concept of its pathophysiology. J Oral Maxillofac Surg.

[9] Vahidi N, Lee TS, Daggumati S, Shokri T, Wang W, Ducic Y (2020). Osteoradionecrosis of the Midface and Mandible: pathogenesis and Management. Semin Plast Surg.

[10] Palma LF, Tateno RY, Remondes CM, Marcucci M, Cortes AR (2020). Impact of radiotherapy on mandibular bone: A retrospective study of digital panoramic radiographs. Imaging Sci Dent.

[11] Demirbaş AK, Ergün S, Güneri P, Aktener BO, Boyacioğlu H (2008). Mandibular bone changes in sickle cell anemia: fractal analysis. Oral Surg Oral Med Oral Pathol Oral Radiol Endod.

[12] Kurşun-Çakmak EŞ, Bayrak S (2018). Comparison of fractal dimension analysis and panoramic-based radiomorphometric indices in the assessment of mandibular bone changes in patients with type 1 and type 2 diabetes mellitus. Oral Surg Oral Med Oral Pathol Oral Radiol.

[13] Şahin O, Odabaşı O, Demiralp KÖ, Kurşun-Çakmak EŞ, Aliyev T (2019). Comparison of findings of radiographic and fractal dimension analyses on panoramic radiographs of patients with early-stage and advanced-stage medication-related osteonecrosis of the jaw. Oral Surg Oral Med Oral Pathol Oral Radiol.

[14] Kato CN, Barra SG, Tavares NP, Amaral TM, Brasileiro CB, Mesquita RA (2020). Use of fractal analysis in dental images: a systematic review. Dentomaxillofac Radiol.

[15] Cordeiro MS, Backes AR, Júnior AF, Gonçalves EH, de Oliveira JX (2016). Fibrous dysplasia characterization using lacunarity analysis. J Digit Imaging.

[16] Basavarajappa S, Konddajji Ramachandra V, Kumar S (2021). Fractal dimension and lacunarity analysis of mandibular bone on digital panoramic radiographs of tobacco users. J Dent Res Dent Clin Dent Prospect.

[17] Silva ME, Santos HS, Ruhland L, Rabelo GD, Badaró MM (2023). Fractal analysis of dental periapical radiographs: a revised image processing method. Oral Surg Oral Med Oral Pathol Oral Radiol.

[18] Uğur Aydın Z, Ocak MG, Bayrak S, Göller Bulut D, Orhan K (2021). The effect of type 2 diabetes mellitus on changes in the fractal dimension of periapical lesion in teeth after root canal treatment: a fractal analysis study. Int Endod J.

[19] Yasar F, Akgünlü F (2005). Fractal dimension and lacunarity analysis of dental radiographs. Dentomaxillofac Radiol.

[20] Kato CN, Barra SG, Abreu LG, Machado VC, Pinheiro JJ, Henriques JA (2022). Fractal analysis of fibrous dysplasia and ossifying fibroma in 2D and 3D CBCT images. J Oral Maxillofac Surg Med Pathol.

[21] Chang CT, Liu SP, Muo CH, Liao YF, Chiu KM, Tsai CH (2022). The impact of dental therapy timelines and irradiation dosages on osteoradionecrosis in oral cancer patients: a population-based cohort study. Oral Oncol.

[22] vElm E, Altman DG, Egger M, Pocock SJ, Gøtzsche PC, Vandenbroucke JP (2007). Strengthening the Reporting of Observational Studies in Epidemiology (STROBE) statement: guidelines for reporting observational studies. BMJ.

[23] White SC, Rudolph DJ (1999). Alterations of the trabecular pattern of the jaws in patients with osteoporosis. Oral Surg Oral Med Oral Pathol Oral Radiol Endod.

[24] Cohen J (1988). Statistical power analysis for the behavioral sciences.

[25] Hajian-Tilaki K (2018). The choice of methods in determining the optimal cut-off value for quantitative diagnostic test evaluation. Stat Methods Med Res.

[26] Youden WJ (1950). Index for rating diagnostic tests. Cancer.

[27] Schisterman EF, Perkins NJ, Liu A, Bondell H (2005). Optimal cut-point and its corresponding Youden Index to discriminate individuals using pooled blood samples. Epidemiology.

[28] Rosenthal JA (1996). Qualitative descriptors of strength of association and effect size. J Soc Serv Res.

[29] Chinn S (2000). A simple method for converting an odds ratio to effect size for use in meta-analysis. Stat Med.

[30] Aarts S, Akker M, Winkens B (2014). The importance of effect sizes. Eur J Gen Pract.

[31] Ren Z, Zhang L, Ding W, Luo Y, Shi Z, Shrestha B (2021). Development and validation of a novel survival model for head and neck squamous cell carcinoma based on autophagy-related genes. Genomics.

[32] Terenzi V, Della Monaca M, Raponi I, Battisti A, Priore P, Barbera G (2020). MRONJ and ORNJ: when a single letter leads to substantial differences. Oral Oncol.

[33] Akashi M, Wanifuchi S, Iwata E, Takeda D, Kusumoto J, Furudoi S (2018). Differences between osteoradionecrosis and medication-related osteonecrosis of the jaw. Oral Maxillofac Surg.

[34] Grisar K, Schol M, Schoenaers J, Dormaar T, Coropciuc R, Vander Poorten V (2016). Osteoradionecrosis and medication-related osteonecrosis of the jaw: similarities and differences. Int J Oral Maxillofac Implants.

[35] Mallya SM, Tetradis S (2018). Imaging of radiation- and medication-related osteonecrosis. Radiol Clin North Am.

[36] Wang TH, Liu CJ, Chao TF, Chen TJ, Hu YW (2017). Risk factors for and the role of dental extractions in osteoradionecrosis of the jaws: a national-based cohort study. Head Neck.

[37] Kuo TJ, Leung CM, Chang HS, Wu CN, Chen WL, Chen GJ (2016). Jaw osteoradionecrosis and dental extraction after head and neck radiotherapy: a nationwide population-based retrospective study in Taiwan. Oral Oncol.

[38] Maesschalck T, Dulguerov N, Caparrotti F, Scolozzi P, Picardi C, Mach N (2016). Comparison of the incidence of osteoradionecrosis with conventional radiotherapy and intensity-modulated radiotherapy. Head Neck.

[39] Saito I, Hasegawa T, Kawashita Y, Kato S, Yamada SI, Kojima Y (2022). Association between dental extraction after radiotherapy and osteoradionecrosis: a multi-centre retrospective study. Oral Dis.

[40] Nabil S, Samman N (2011). Incidence and prevention of osteoradionecrosis after dental extraction in irradiated patients: a systematic review. Int J Oral Maxillofac Implants.

[41] Marx RE, Johnson RP (1987). Studies in the radiobiology of osteoradionecrosis and their clinical significance. Oral Surg Oral Med Oral Pathol.

[42] Fujita M, Tanimoto K, Wada T (1986). Early radiographic changes in radiation bone injury. Oral Surg Oral Med Oral Pathol.

[43] Chan KC, Perschbacher SE, Lam EW, Hope AJ, McNiven A, Atenafu EG (2016). Mandibular changes on panoramic imaging after head and neck radiotherapy. Oral Surg Oral Med Oral Pathol Oral Radiol.

[44] Kluth EV, Jain PR, Stuchell RN, Frich JC (1988). A study of factors contributing to the development of osteoradionecrosis of the jaws. J Prosthet Dent.

[45] Santolia D, Dahiya S, Sharma S, Khan MA, Mohammed N, Priya H (2022). Fractal Dimension and radiomorphometric analysis of orthopanoramic radiographs in patients with tobacco and areca nut associated oral mucosal lesions: A pilot in-vivo study in a North Indian cohort. Oral Surg Oral Med Oral Pathol Oral Radiol.

[46] Franciotti R, Moharrami M, Quaranta A, Bizzoca ME, Piattelli A, Aprile G (2021). Use of fractal analysis in dental images for osteoporosis detection: a systematic review and meta-analysis. Osteoporos Int.

